# Reading Margery Kempe’s inner voices

**DOI:** 10.1057/s41280-017-0051-5

**Published:** 2017-06

**Authors:** Corinne Saunders, Charles Fernyhough

**Affiliations:** a Department of English Studies, Durham University, Durham, UK; b Department of Psychology, Durham University, Durham, UK

## Abstract

This article draws on research from the major collaborative research project *Hearing the Voice*, based at Durham University, to reconsider and foreground Margery Kempe’s inner voices, and hence, to return to an emphasis on inner, spiritual experience as shaping her *Book*. The richness of Margery’s multi-sensory experience, and the care with which it is depicted, is illuminated by and illuminates the experience of contemporary voice-hearers, offering a powerful alternative perspective to often reductive bio-medical understandings. Contemporary cognitive frameworks, particularly scientific accounts of inner speech, are in turn employed to open out Margery’s inner voices and to offer insights into the psychology of spiritual meditation.

‘It is time to read Margery Kempe’s inner voices as a projection of her own spiritual understanding of divine interaction with her, and hence as an insight into her own mentality,’ writes Barry Windeatt (Windeatt, 2004, 15). Margery’s spiritual life has been controversial since the rediscovery of her *Book* in 1934, which quickly demonstrated that she was far from the ‘devout ancress’ that Henry Pepwell had described her as when reprinting Wynkyn de Worde’s pamphlet based on her book in 1521 (Kempe, 2004, 1). The book evoked responses emphasising Margery’s neurosis and hysteria, until it was reclaimed by feminist readers celebrating her public voice and actions; recent studies have gone further, emphasising her radical Christianity.^1^ Once seen as a mystic, she is now viewed as a dissenter. Windeatt’s call for a new approach challenges current trends, evident in *The Companion to the Book of Margery Kempe* (2004) that Windeatt introduces. The *Companion* focuses on the *Book*’s engagement with the socio-cultural and political currents of its time: Margery’s compulsive ‘cryings,’ once the mark of hysteria, are interpreted as performing piety, unusual behaviours ‘picked from a menu of practices’ (Salih, 2004, 176). In moving to an almost exclusively historicist approach, scholarship has found a way of normalising and authorising Margery and her *Book* – but in the process has sidelined the inner, spiritual experience that is so powerfully written on Margery’s body and into her narrative.

Our participation in the interdisciplinary project *Hearing the Voice* has led us to take up Windeatt’s challenge to listen to Margery’s inner voices. The project (based at Durham University and funded by the Wellcome Trust) explores the phenomenon of hearing voices without external stimuli (auditory verbal hallucinations), and it has drawn attention to the relevance of premodern narratives for voice-hearers in the present, and to the correspondences as well as differences between past and present. Although auditory-verbal hallucinations can be, and are typically assumed to be, symptoms of psychosis, they are experienced by a significant proportion of the ‘healthy’ population (large-scale studies suggest approximately one percent, and higher percentages for more fleeting experiences), and while frequently distressing, they can also be benign or positive experiences (McCarthy-Jones, 2012, 170–88). Margery’s narrative offers perspectives on such experiences other than bio-medical explanations, perspectives that can be enabling and powerfully resonant. The *Book*’s recognition of the challenge of placing abnormal experience has an immediate resonance for modern voice-hearers, who are faced with trying to find an explanatory frame for the inexplicable, intrusive, and sometimes terrifying voices they hear, and for other kinds of unusual experience – the sense, for example, of an invisible ‘felt presence.’^2^ While it would be simplistic to take her *Book* simply as a record of experience (a case history), it conveys a powerful impression of her ‘shaping imagination’ (Windeatt, 2004, x) and it is, above all, an account of ‘hyr felyngys and hir revelacyons’ (78–80). In its deep engagement with vernacular devotional writings such as those of Richard Rolle and Walter Hilton, and with the lives of holy women such as Bridget of Sweden and Mary of Oignies, as well as through Margery’s working relationship with her amanuensis, the *Book* becomes a polyphony of voices, evoking and commenting on lived experience and the difficulty of understanding that experience.

If we are to return to foregrounding Margery’s voices, how can we best approach them? Her experiences and behaviours have attracted a variety of medical diagnoses, ranging from hysteria to psychosis to temporal lobe epilepsy.^3^ While her early illness can persuasively be placed as post-natal psychosis, and while some of her unusual experiences may have had physiological causes, these bio-medical models, which replace the explanatory frame of the supernatural with the language of delusion and hallucination, are reductive: they do not reflect Margery’s or her contemporaries’ understandings of her experiences and may, indeed, render them more alien. Contemporary non-medical accounts of voice-hearing and unusual experience in the healthy population provide closer analogues, particularly accounts of religious experience in evangelical communities and in non-Western tradition.^4^ Margery’s unusual experiences need to be approached as aspects of her spiritual life, in keeping with the writings that shaped her individual piety. They are in keeping too with medieval concepts of thought and sensory perception, which allowed for the notion of an inner eye and ear, and hence the possibility of visionary experience and of hearing inner voices. Qualitative studies have shown that supernatural or spiritual explanations remain some of the most available and powerful for voice-hearers themselves.^5^

But Margery’s experiences also differ from contemporary accounts of voice-hearing in their frequently integrated multi-sensory quality. In medieval writing more generally, it is unusual to find ‘voice-hearing’ – the experience foregrounded today – treated in isolation. Margery’s *Book* vividly depicts the multi-sensory nature of vision: she is cured from her madness by the appearance of Jesus, ‘in lyknesse of a man, most semly, most bewtyvows, and most amyable, [. . .] clad in a mantyl of purpyl sylke, syttyng upon hir beddys syde’ (227, 230).^6^ Her homely visions of Christ as lover are opposed by grotesquely sexual, demonic visions, signalling the difficulty of chastity. Like Julian of Norwich, she emphasises the ‘gostly’ [spiritual] eye: this inward ‘seeing,’ however, involves a range of inner senses (Julian of Norwich, 2016). Margery enters into a three-dimensional spiritual world, where she participates in biblical scenes relating to the life of Christ, from the birth of the Virgin to the Nativity to the Passion itself: she looks after the child Mary, swaddles the infant Jesus, and consoles the Virgin with ‘a good cawdel’ after the Crucifixion (6561). Her feminine roles of mother and housekeeper are enacted within the world of the inward eye and, again and again, she reiterates that what she sees with that eye surpasses that which is seen by the bodily eye.

The powerfully multi-modal quality of Margery’s experience in many ways was crucial to its impact on her society.^7^ Yet the voice, heard within the soul or mind, remains preeminent across the book. References to speech, words, and conversation with the Lord occur again and again (as when she recalls His direct question, ‘What seyst thu, Margery, to my Fader of thes wordys that he spekyth to the,’ 2840–41). The *Book*’s special emphasis is on the ‘wonderful spechys and dalyawns [conversations] whech owr Lord spak and dalyid to hyr sowle’ (52–53). Such ‘dalliance’ ‘ravishes’ the spirit. The term ‘dalliance’ may be used of serious or moral conversation, but may also suggest intimacy through its connections with social converse and amorousness. Margery converses extensively too with Mary, and is instructed to receive the apostles, Mary Magdalene, and other saints within her soul.

As with Margery’s multi-modal experiences, hearing the Lord’s voice requires active participation: it is ‘in gret rest of sowle a gret whyle’ that she has ‘hy contemplacyon day be day, and many holy spech and dalyawns of owyr Lord Jhesu Cryst both afornoon and aftyrnoon’ (924–926). She pleases the Lord by being ‘in silens’ and allowing him to ‘speke in [her] sowle’ (2922–2923), and she is loath to care for her ailing husband because she will be unable to attend (‘tendyn,’ 6053) to the Lord, an attention most often enacted through prayer in church. Narrating the affects of ‘dalyawnce’ (2187) repeatedly requires sensory metaphors, especially those of taste (‘swetnesse,’ 2189), and stimulates other affective experiences – ‘swet terys of hy devocyon’ (927), the fire or flame of love (2894), and physical ‘fallyng’ (2190). But the narrative is also distinguished by a sense of the impossibility of conveying the ineffable, and the gap between language and experience. The *Book* is dominated by the sound of Margery’s cryings, the voice of vision, reflecting the impossibility of fully articulating vision in language. Words gesture towards but never entirely capture feeling, always at a remove from her experiences, ‘so hy abovyn hir bodily wittys that sche myth nevyr expressyn hem wyth hir bodily tunge liche as sche felt hem’ (6793–6795). What she hears is repeatedly characterised as mysterious, mystical, ‘secretys of hir sowle’ (1064) and as ‘aboven hyr reson’ (62). Margery seems to probe the nature of voice-hearing itself as she describes to the English friar whom she meets at Assisi ‘hir maner levyng, of hir felingys, of hir revelacyons, and of the grace that God wrowt in hir sowle be holy inspiracyons and hy contemplacyons, and how owyr Lord dalyed to hir sowle *in a maner of spekyng*’ (2575–2578, our italics). The phrase chimes closely with descriptions by voice-hearers of the voices they seem to hear within the mind.

The voices Margery hears are not exclusively interior, and the book suggests an acute awareness of different kinds of hearing: lying in bed, she hears ‘wyth hir bodily erys a lowde voys clepyng: “Margery”’; on waking, God speaks directly to her, ‘Dowtyr’ (4381, 4386). She is also alert to different identities of the voices she hears. When she is sinful, the Lord’s speech is denied her, replaced by demonic voices and ‘horybyl syghtys and abhominabyl’ (4863–4864): the devil ‘bad hir in hir mende’ to choose which man she will prostitute herself with (4869–4870). Her prayer summons ‘hir good awngel’ (4887) who explains that her lack of faith will be punished for twelve days by the withdrawal of the Lord’s voice; her return to grace is marked by His speaking to her once again. The book is shaped most of all by these inner conversations with the Lord, sometimes very extended (such as the late disquisitions on charity and love and on heavenly reward). The Lord’s voice is by no means always presented in terms of spiritual ravishment, but instead frequently figures as a familiar aspect of Margery’s mind, offering her guidance in her day to day decisions and a running commentary of a dialogic, sometimes even mundane, kind – very like the experiences of some voice-hearers today. The voice is deeply practical: the Lord offers advice on where Margery should go, to whom she should speak and what she should say (particularly when it is confrontational), how she should treat her husband, what ascetic practices she should adopt and when, how she should dress, and whether she should write the book. He assures her of her well-being, safety, and health, and those of the people around her, and provides explanations for natural events, for the responses of people to her, and for her own physical states – illness, pain, the affects of vision. This conversational mode – by contrast to multi-sensory vision – is present throughout, but becomes more prominent later in the narrative.

Sounds more generally, mainly represented as exterior hallucinations, are a special aspect of God’s teaching. The experience that converts Margery to a life of chastity, purgation, and prayer, is auditory, ‘a sownd of melodye so swet and delectable, hir thowt, as she had ben in paradyse’ (325–326). Later, ‘so hedows a melodye that sche mygth not ber it’ (1242) causes her to faint, and she hears ‘wyth hir bodily erys sweche sowndys and melodiis that sche myth not wel heryn what a man seyd to hir in that tyme, les he spoke the lowder’ (2868–2870), as well as ‘gret sowndys and gret melodiis wyth hir bodily erys’ that signal heavenly merriment (6224–6245). Her revelatory experience is characterised by a diversity of sounds:

Thys creatur had divers tokenys in hir bodily heryng. On was a maner of sownde, as it had ben a peyr of belwys blowyng in hir ere. Sche, beyng abasshed therof, was warnyd in hir sowle no fer to have, for it was the sownd of the Holy Gost. And than owr Lord turnyd that sownde into the voys of a dowe, and sithyn he turnyd it into the voys of a lityl bryd whech is callyd a reedbrest, that song ful merily oftyntymes in hir ryght ere. And than schuld sche evyrmor han gret grace aftyr that sche herd swech a tokyn. And sche had been used to swech tokenys abowt xxv yer at the writyng of this boke. (2965–2974)

The phenomenological richness of Margery’s experiences is congruent with what is known about the experience of hearing voices, both by those meeting the criteria for psychiatric diagnoses and by that significant minority of individuals for whom hearing voices is part of everyday experience. While voice-hearing has come to be prioritised as a symptom, the phenomenological range of Margery’s experiences – from clear external speech to the susurrus of the Holy Ghost to the songs of birds – reflects that described by many voice-hearers today, as does the multi-sensory nature of Margery’s experiences. Although truly ‘fused’ hallucinations (in which the same entity is, for example, both seen and heard) are rare in contemporary reports (Waters et al., 2014), many voices-hearers report that their experiences are accompanied by sensory and bodily sensations (Woods et al., 2015).

Windeatt’s call for a refreshed approach to Margery’s inner voices also invites us to consider them specifically as a dialogue – a kind of internal conversation. This view resonates excitingly with current scientific accounts of inner speech, the conversation with the self that many people report when they reflect on their inner experience. In the Dialogic Thinking model, inspired by the writings of the Russian psychologist L. S. Vygotsky ([1934] 1987), inner speech develops through the gradual internalisation of social exchanges and thus retains its dialogic character. This view has been supported by questionnaire studies asking people about the quality of their inner speech (McCarthy-Jones and Ferny-hough, 2011) and by recent neuroimaging evidence showing that social cognition (or ‘theory of mind’) networks are involved when participants conduct an internal conversation with themselves (Alderson-Day et al., 2016).^8^

Another important feature of inner dialogue concerns the extent to which it is abbreviated or condensed relative to external speech.^9^ The Dialogic Thinking model holds that inner speech can vary between expanded and condensed forms: the former representing cases where inner dialogue takes the form of full sentences retaining the to-and-fro of conversation, the latter describing exchanges in which the linguistic properties of the utterances are largely stripped away (approaching the state described by Vygotsky as ‘thinking in pure meanings’ [Vygotsky, [1934] 1987, 281]). Auditory verbal hallucinations, or voice-hearing experiences, are proposed to result when condensed inner speech is temporarily re-expanded into an expanded inner dialogue, resulting in the sudden blooming into consciousness of multiple voices (Fernyhough, 2004).

Thinking about Margery Kempe’s experiences as inner dialogues presents a spur to the further development of the Dialogic Thinking model. That is, we can consider Margery’s experiences as representing two forms of inner dialogue in which God (or one of the other spiritual entities that inhabit her Book) occupies the ‘open slot,’ thus adopting the role of internal interlocutor. In its condensed form, Margery’s internal dialogue is a type of inner conversation whose linguistic features are minimised and in which the distinct perspectives in the dialogue are represented simultaneously: a state of ‘being with’ God. In its expanded form, Kempe’s inner dialogue is an explicit conversation with God in which the to-and-fro of external conversation is preserved. Margery hears God speaking to her as an interlocutor, and she speaks back.

Aligning Margery Kempe with the phenomenon of voice hearing gives us a radically new perspective on the psychology of spiritual meditation. It demonstrates how meeting Windeatt’s challenge can present us with a frame of reference that both sheds new light on the text and offers provocative ways of thinking about the cognitive processes evident in (to quote Windeatt again) ‘a praying mind talking to itself’ (Windeatt, 2014, n.p.).

## Footnotes

1 See, for example, Staley (1994).

2 See Alderson-Day (2016).

3 See Lawes (1999) and, on the limits of these diagnoses, Torn (2011a, 2011b).

4 See Luhrmann (2012).

5 See, for example, Conor McCormack’s film *In the Real* (2015), which explores the experiences of members of the Bristol Hearing Voices Network, including the vivid account by one voice-hearer, Errol, of his experience of the spirit world around him. See also Cook (2015) and Dein and Cook (2015).

6 This and all following quotations from the *Book of Margery Kempe* are taken from Windeatt’s edition (Kempe, 2004) and are indicated by line number.

7 See Fernyhough (2016, 155–6).

8 For further studies of internal dialogue, including its social nature and structure, see Alderson-Day and Fernyhough (2015) and Fernyhough (2016). On the identity of the interlocutor in internal dialogue see Peirce (1933), Luria (1966), Holquist (1990), Fernyhough (1996, 2010).

9 See Vygotsky ([1934] 1987), Fernyhough (2004).

## References

[R1] Alderson-Day B (2016). The Silent Companions. The Psychologist.

[R2] Alderson-Day B, Fernyhough C (2015). Inner speech: Development, cognitive functions, phenomenology, and neurobiology. Psychological Bulletin.

[R3] Alderson-Day B, Weis S, McCarthy-Jones S, Moseley P, Smailes D, Fernyhough C (2016). The Brain’s Conversation with Itself: Neural Substrates of Dialogic Inner Speech. Social Cognitive & Affective Neuroscience.

[R4] Cook CCH (2015). Religious Psychopathology: The Prevalence of Religious Content of Delusions and Hallucinations in Mental Disorder. International Journal of Social Psychiatry.

[R5] Dein S, Cook CCH (2015). God Put a Thought into My Mind: The Charismatic Christian Experience of Receiving Communications from God. Mental Health, Religion & Culture.

[R6] Fernyhough C (1996). The dialogic mind: A dialogic approach to the higher mental functions. New Ideas in Psychology.

[R7] Fernyhough C (2004). Alien voices and inner dialogue: Towards a developmental account of auditory verbal hallucinations. New Ideas in Psychology.

[R8] Fernyhough C, Sokol B, Müller U, Carpendale J, Young A, Iarocci G (2010). Vygotsky, Luria and the Social Brain. Self- and Social-Regulation: Exploring the Relations between Social Interaction, Social Cognition, and the Development of Executive Functions.

[R9] Fernyhough C (2016). The Voices Within: The History and Science of How We Talk to Ourselves.

[R10] Holquist M (1990). Dialogism: Bakhtin and His World.

[R11] Windeatt B, Julian of Norwich (2016). Revelations of Divine Love.

[R12] Kempe M, Windeatt B (2004). The Book of Margery Kempe.

[R13] Lawes R, Glasscoe M (1999). The Madness of Margery Kempe. The Medieval Mystical Tradition: England, Ireland, and Wales.

[R14] Luhrmann TM (2012). When God Talks Back: Understanding the American Evangelical Relationship with God.

[R15] Luria AR (1966). Higher Cortical Functions in Man.

[R16] McCarthy-Jones S (2012). Hearing Voices: The Histories, Causes and Meanings of Auditory Verbal Hallucinations.

[R17] McCarthy-Jones SR, Fernyhough C (2011). The varieties of inner speech: Links between quality of inner speech and psychopathological variables in a sample of young adults. Consciousness and Cognition.

[R18] McCormack C (2015). In the Real.

[R19] Peirce CS, Hartshorne C, Weiss P (1933). Collected Papers of Charles Sanders Peirce.

[R20] Salih S, Arnold JH, Lewis KJ (2004). Margery’s Bodies: Piety, Work and Penance. A Companion to the Book of Margery Kempe.

[R21] Staley L (1994). Margery Kempe’s Dissenting Fictions.

[R22] Torn A (2011a). Looking Back: Medieval Mysticism or Psychosis. The Psychologist.

[R23] Torn A (2011b). Madness and Mysticism: Can a Mediaeval Narrative Inform our Understanding of Psychosis?. History and Philosophy of Psychology.

[R24] Vygotsky L, Rieber RW, Carton AS, Minick N ([1934] 1987). Thinking and Speech. The Collected Works of L.S. Vygotsky.

[R25] Waters F (2014). Visual Hallucinations in the Psychosis Spectrum and Comparative Information From Neurodegenerative Disorders and Eye Disease. Schizophrenia Bulletin.

[R26] Windeatt B, Arnold JH, Lewis KJ (2004). Introduction: Reading and Re-reading The Book of Margery Kempe. A Companion to the Book of Margery Kempe.

[R27] Windeatt B (2014). Shown Voices: Voices as Vision in Some English Mystics.

[R28] Woods A, Jones N, Alderson-Day B, Callard F, Fernyhough C (2015). Experiences of Hearing Voices: Analysis of a Novel Phenomenological Survey. Lancet Psychiatry.

